# Young Bone-Marrow Sca-1^+^ Stem Cells Rejuvenate the Aged Heart and Improve Function after Injury through PDGFRβ-Akt pathway

**DOI:** 10.1038/srep41756

**Published:** 2017-01-31

**Authors:** Shu-Hong Li, Lu Sun, Lei Yang, Jiao Li, Zhengbo Shao, Guo-Qing Du, Jun Wu, Richard D. Weisel, Ren-Ke Li

**Affiliations:** 1 Division of Cardiovascular Surgery, Toronto General Research Institute, University Health Network, Toronto, ON, Canada; 2 Research Institute, Second Affiliated Hospital of Harbin Medical University, Harbin, China; 3 Department of Cardiology, Second Affiliated Hospital of Guangzhou Medical University, Guangzhou Institute of Cardiovascular Disease, Guangzhou, China; 4 Division of Cardiac Surgery, Department of Surgery, University of Toronto, Toronto, ON, Canada

## Abstract

Bone marrow (BM) reconstitution with young BM cells in aged recipients restores the functionality of cardiac resident BM-derived progenitors. This study investigated the cell type primarily responsible for this effect. We reconstituted old mice with BM cells from young or old mice and found that the number of stem cell antigen 1 (Sca-1) cells homing to the heart was significantly greater in young than old chimeras. We then reconstituted old mice with young BM Sca-1^+^ or Sca-1^−^ cells. We found that Sca-1 cells repopulated the recipient BM and homed to the heart. The number of BM-derived cells in the aged myocardium co-expressing PDGFRβ was 3 times greater in Sca-1^+^ than Sca-1^−^ chimeric mice. Sca-1^+^ chimeras had more active cell proliferation in the infarcted heart and improved ventricular function after MI. The improved regeneration involved activation of the PDGFRβ/Akt/p27^Kip1^ pathway. Sca-1^+^ stem cells rejuvenated cardiac tissue in aged mice. Restoration of the Sca-1^+^ subset of stem cells by BM reconstitution improved cardiac tissue regeneration after injury in aged mice.

Aging is associated with an impairment of endogenous stem and progenitor cells, including cardiac progenitor cells, which may contribute to the limited regenerative capacity of the aged heart^1^,^2^,^3^. After a myocardial infarction (MI), regenerative cells from the bone marrow (BM) and heart are recruited to the site of injury for repair^4^,^5^. We and others have shown that aging reduces such cell recruitment^3^,^6^,^7^, thereby compromising intrinsic cardiac repair^8^,^9^. While previous studies have suggested that the age of the entire stem cell pool negatively impacts cardiac regeneration, we recently determined that the age of a specific pool of stem cells, the cardiac-resident BM-derived progenitor cells, had the biggest impact on cardiac recovery after MI in aged animals^10^. While this work has established that BM reconstitution can facilitate stable integration of young progenitor cells into the myocardium of aged recipients and restore the cardiac regenerative capacity of aged individuals, the BM cell type primarily responsible for this effect was not identified.

Stem cell antigen 1 (Sca-1) is an 18-kDa glycosyl phosphatidylinositol-anchored protein (GPI-AP) that was originally identified as an antigen upregulated in activated lymphocytes in mice^11^. It belongs to the lymphocyte-activation protein-6 (Ly-6) family, whose function still remains to be clarified. Although Sca-1 has been widely used as a marker to isolate hematopoietic stem cells, it is also expressed by a variety of stem, progenitor, and differentiated cell types in many tissues and organs^12^. Sca-1 expression has been identified in putative stem/progenitor cell populations within the skeletal system^13^, mammary gland^14^, prostate^15^, dermis^16^, skeletal muscle^17^, and liver^18^. The functions of Sca-1 include the promotion of cell adhesion and proliferation that are critical for optimal hematopoietic activity^12^.

Sca-1 has been used as a surrogate marker to identify cardiac stem cells in the heart^19^. The functional importance of Sca-1 under pathological conditions has been extensively evaluated. It has been shown that lack of Sca-1 in the adult mouse heart results in minor developmental contractile defects as well as age-associated hypertrophy^20^. Cardiac overexpression of Sca-1 significantly attenuated cardiac hypertrophy and fibrosis under conditions of pressure overload, whereas cardiac function was preserved^21^. Conversely, Sca-1 disruption aggravated cardiac hypertrophy, fibrosis, and dysfunction after aortic banding injury^21^. These results suggest that Sca-1 deficiency promoted cardiac dysfunction in response to pressure overload involving uncontrolled precursor recruitment and exhaustion of the precursor pool^21^.

Isolated Sca-1 cells have the capacity to home to the heart after intravenous injection into either neonates^19^ or adult mice following MI^22^. Furthermore, Sca-1 expression appears to play a role in the expansion and survival of cardiac progenitor cells in the infarcted myocardium^23^. After injury, the number of Sca-1^+^ cells increases in the myocardium^24^, and progenitor cells from BM migrate to the myocardium to facilitate repair^25^. This suggests that Sca-1 cells contribute to regeneration and repair after an MI.

Here, we conducted two studies. Study 1: Using whole BM reconstitution, we identified the Sca-1^+^ cell as the young BM cell type that had the greatest ability to home to the myocardium of the aged recipient mouse. Study 2: To investigate the effects of Sca-1^+^ cells on rejuvenation of the aged heart, we isolated Sca-1^+^ or Sca-1^−^ cells from the BM of young donor mice and infused them into lethally-irradiated old recipients to generate Sca-1^+^ or Sca-1^−^ chimeras, respectively. We found that BM chimerism established with young Sca-1^+^ cells was associated with better restoration of myocardial progenitors and improved healing of the aged heart after MI.

## Results

### Young BM Sca-1^+^ cells had the greatest ability to migrate to the aged myocardium at steady-state

**Figure 1:**
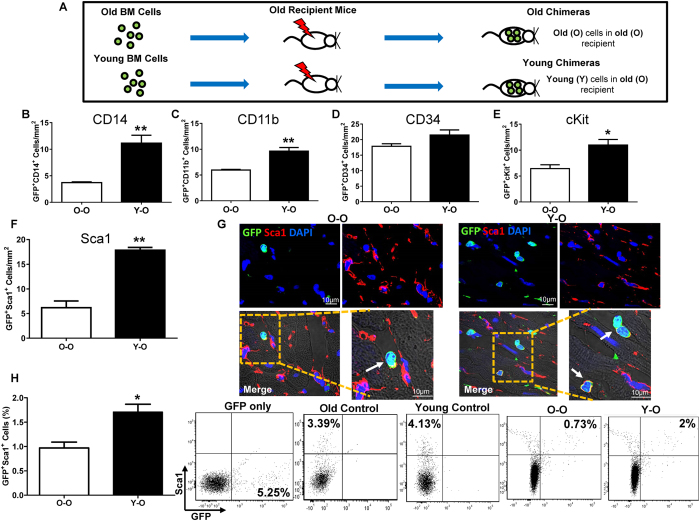
Young BM Sca-1^+^ cells had the greatest ability to migrate to the aged myocardium at steady-state after whole BM reconstitution. Bone marrow (BM) cells from old (O) or young (Y) GFP mice were used to reconstitute the BM of lethally-irradiated old mice, generating old (O-O) and young (Y-O) chimeras **(A**). Progenitor cells in the hearts of recipients were evaluated 12 weeks later. Quantification of GFP/CD14 (**B**, n = 3/group), GFP/CD11b **(C**, n = 3/group), GFP/CD34 (**D**, n = 3/group), GFP/cKit **(E**, n = 4/group), and GFP/Sca-1 **(F**, n = 4/group) double-positive cells in Y-O and O-O chimeric hearts. Immunolabeling of myocardial sections for Sca-1 and GFP (**G**). Arrow indicates representative cells positive for GFP and Sca-1. GFP^+^/Sca-1^+^ cells quantified by flow cytometry in the Y-O and O-O chimeric hearts **(H**, n = 3/group, **p < 0.01, *p < 0.05). Data analysis used un-paired *t*-test (**B**,**C**,**D**,**E**,**F**,**H**). Data shown are mean ± SEM.

Whole BM cells from old (O) or young (Y) GFP^+^ mice were used to reconstitute the BM of lethally-irradiated old mice, generating old (O-O) and young (Y-O) chimeras (Fig. 1A). Mice were sacrificed 12 weeks after BM reconstitution for immunofluorescent staining and flow cytometric analysis to identify homed BM progenitors. Immunohistochemistry was performed using an array of progenitor cell markers to compare the number of homed progenitors in the aged heart at steady state after BM reconstitution. The number of homed CD14^+^ (Fig. 1B) and CD11b^+^ (Fig. 1C) cells, which represents total monocytic progenitors, was significantly greater in Y-O than O-O chimeras. The inability of old BM cells to home to the heart implies that it may be a dysfunctionality of these cells which led to this effect. Indeed, p16 mRNA expression was significantly higher in old than young BM cells. Moreover, immunostaining of the p16 protein showed that there were more p16^+^ cells in old than young BM cells (unpublished observation). The number of homed BM CD34^+^ progenitors (Fig. 1D) was not significantly different between the two chimeric groups. The number of homed BM cKit^+^ (Fig. 1E) and Sca-1^+^ progenitors (Fig. 1F and G) was significantly higher in Y-O than O-O, but the magnitude of increase was much higher for homed Sca-1^+^ (1.88 fold) than cKit^+^ (0.71 fold) progenitors. This result was further confirmed by flow cytometric analysis (Fig. 1H) which found that the number of Sca-1/GFP double-positive cells was nearly doubled in Y-O compared to O-O chimeric hearts. Since the greatest difference between young and old migratory cells was in the Sca-1^+^ cell population, we further investigated the effects of Sca-1^+^ cells on rejuvenation of the aged heart at steady-state.

### Sca-1^+^ chimeras restored progenitors in the aged heart

**Figure 2:**
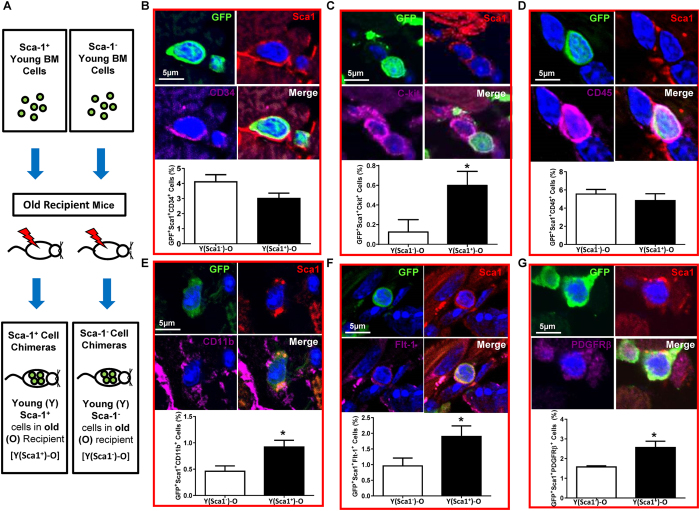
Homed BM Sca-1 cells maintain monocyte/progenitor cell pool in aged heart. Sca-1^+^ or Sca-1^−^ bone marrow (BM) cells from young (Y) GFP mice were used to reconstitute old mice, generating Sca-1^+^ [Y(Sca1^+^)-O] and Sca-1^−^ [Y(Sca1^−^)-O] cell chimeras, respectively **(A)**. Progenitor cells in the hearts of recipients were evaluated 12 weeks later. Characterization and quantification by immunolabeling of myocardial sections for Sca-1/GFP and CD34 (**B**), cKit **(C**), CD45 **(D)**, CD11b **(E**), Flt-1 **(F**), and PDGFRβ (**G**) triple-positive cells. Representative confocal images. n = 3/group; *p < 0.05. Data analysis used un-paired *t*-test (**B**,**C**,**D**,**E**,**F**,**G**). Data shown are mean ± SEM.

To determine the reparative capacity of the BM Sca-1 subset of cells in aged mice, Sca-1^+^ cells isolated from young (Y) GFP^+^ mice were used for reconstitution of lethally-irradiated old (O) recipients to generate Sca-1^+^ chimeras [Y(Sca1^+^ )-O]. Sca-1^−^ cells from the same donors were used to generate Sca-1^−^ chimeras [Y(Sca1^−^)-O] as a control (Fig. 2A). Twelve weeks after reconstitution, the Sca-1^+^ subset had more effectively replaced the stem and progenitor cells in the BM and blood of aged recipients than the Sca-1^−^ subset (*p* < 0.05 for all groups; Supplementary Fig. S1). A similar response was seen in the heart. Confocal imaging revealed that some of the cardiac-resident BM-derived progenitor cells were positive for myeloid markers CD34 (Fig. 2B), CD117 (cKit, Fig. 2C), CD45 (Fig. 2D), and CD11b (Fig. 2E). The number of CD34^+^ or CD45^+^ cells was similar in the aged hearts of Sca-1^+^ and Sca-1^−^ chimeras. However, Sca-1^+^ chimeras had significantly more cKit^+^ (Fig. 2C) and CD11b^+^ (Fig. 2E) cells. Some of the cardiac-resident BM-derived progenitor cells were also positive for Flt-1, an endothelial progenitor marker (Fig. 2F), and PDGFRβ, a pericyte marker (Fig. 2G). The number of Flt-1^+^ and PDGFRβ^+^ cells was significantly higher in Sca-1^+^ than Sca-1^−^ chimeric hearts. These BM progenitors were often located within areas with a high density of fibronectin near other non-BM cells, and they had a high nuclear-to-cytoplasmic ratio (Fig. 2). This result indicates that the Sca-1^+^ cell reconstitution repopulated stem/progenitor cells in aged recipient hearts.

### Healing of the aged heart was improved in Sca-1^+^ chimeras

**Figure 3:**
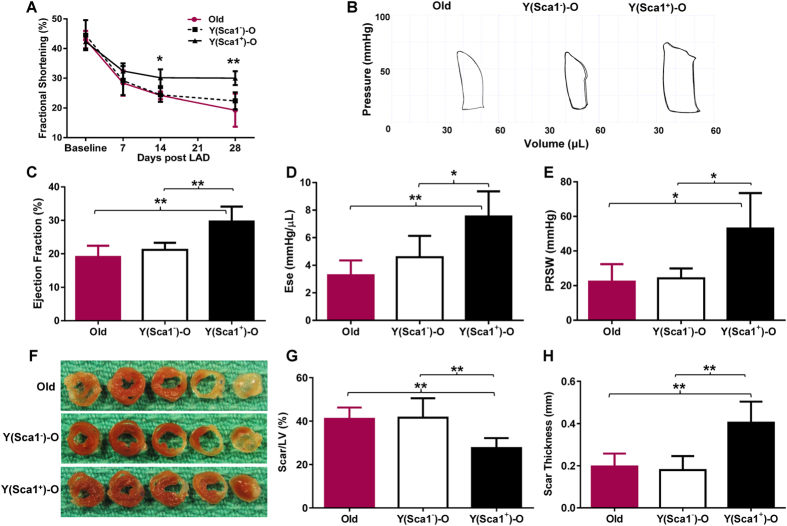
Rejuvenation of BM-derived cardiac resident Sca-1^+^ cells associated with improved healing of aged heart. The left anterior coronary artery of Sca-1^+^ [(YSca1^+^)-O] and Sca-1^−^ [(YSca1^−^)-O] chimeric mice and aged control (Old) mice was ligated 12 weeks after bone marrow reconstitution. Ventricular function was evaluated by serial echocardiography (n = 5–6/group) 4 weeks post-myocardial infarction (MI) with fractional shortening **(A**). (**B**) Representative pressure-volume (P-V) loops from each of the three groups. Invasive haemodynamic measurements (n = 4–6/group) taken 4 weeks post-MI were used to calculate load-dependent indices of ventricular function, including ejection fraction (**C**). Load-independent indices including end-systolic elastance [Ese] **(D)** and preload recruited stroke work [PRSW] (**E**) were also measured. Representative whole hearts sectioned transversely at the mid-papillary muscle level (**F**) were used to quantify scar area (**G**) and thickness (**H**, n = 5–6/group) 4 weeks post–MI. *p < 0.05, **p < 0.01. LAD: ligation of left anterior coronary artery. Data analysis used one- way repeated-measures ANOVA followed by Bonferroni *post-hoc* tests for multiple comparisons (**A**) and one-way ANOVA followed by Tukey *post-hoc* tests to evaluate differences among groups (**C**,**D**,**E**,**G**,**H**). Data shown are mean ± SEM.

MI (ligation of left anterior coronary artery) was induced in Sca-1 chimeras (twelve weeks after generating chimeras using Sca-1 BM reconstitution), and we evaluated cardiac function following MI for four weeks. Fractional shortening was similar in Sca-1^−^ chimeras and aged control mice (Old) that did not receive any BM reconstitution (Fig. 3A). In contrast, fractional shortening in Sca-1^+^ chimeras was significantly improved relative to Sca-1^−^ chimeras beginning 2 weeks after MI (Fig. 3A). Representative pressure-volume (P-V) loops from each of the three groups are shown in Fig. 3B. Invasive haemodynamic measurements taken 4 weeks post-MI showed that Sca-1^+^ chimeras had a significantly greater ejection fraction than Sca-1^−^ chimeras and Old mice (Fig. 3C) which was sustained for up to 4 months (Supplementary Fig. S2). The same pattern was found for other load-dependent indices of cardiac function (Supplementary Fig. S3). The load-independent indices end-systolic elastance (Fig. 3D) and preload recruited stroke work (Fig. 3E) were significantly higher in Sca-1^+^ than Sca-1^−^ and Old mice when measured 4 weeks post-MI. The infarct scar was also thicker and less extensive after recovery (4 weeks post-MI) in Sca-1^+^ chimeric hearts (Fig. 3F, G and H).

### Homed BM Sca-1^+^ cells stimulated proliferation of progenitor cells after MI

**Figure 4:**
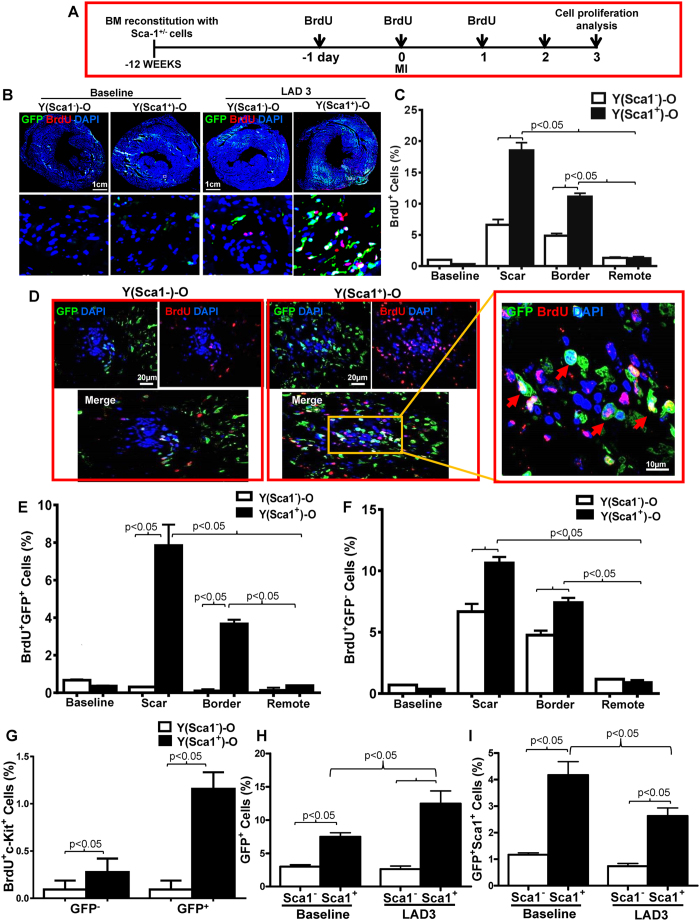
Homed BM Sca-1^+^ cells stimulated proliferation of donor and host progenitor cells after MI. (**A**) Coronary artery ligation was performed 12 weeks after the bone marrow (BM) reconstitution of old recipients with young Sca-1^+^ or Sca-1^−^ cells, and BrdU was given 1 day prior to myocardial infarction (MI), on the day of MI, and 1 day after MI. Cardiac proliferating cells were assessed 3 days post-MI (LAD3). Immunolabeling of myocardial sections permitting identification of cells which were either GFP^+^ (young BM cells; green) and/or BrdU^+^ (proliferating cells; red). Nuclei are stained blue with DAPI (**B**). Quantification of BrdU^+^ cells in the scar, border, and remote regions of the Sca-1^+^ [(YSca1^+^)-O] and Sca-1^−^ [(YSca1^−^)-O] chimeric hearts (**C**, n = 3/group). Representative confocal images of BrdU^+^/GFP^+^ donor cells in the scar region **(D)** and quantification of BrdU^+^/GFP^+^ donor cells (**E**, n = 3/group) and BrdU^+^/GFP^−^ host cells (**F**, n = 3/group) in the scar, border, and remote regions of chimeric hearts. Quantification of BrdU^+^/cKit^+^ cells in the GFP^+^ and GFP^−^ fraction (**G**, n = 3/group) in the scar, border, and remote regions of the Sca-1^+^ and Sca-1^−^ chimeric hearts. GFP^+^ (**H**, n = 4/group) and GFP^+^/Sca-1^+^ cells (**I**, n = 3–5/group) quantified by flow cytometry in chimeric hearts at baseline and 3 days post-MI. BrdU: 5-bromo-2′-deoxyuridine; DAPI: 4′,6-diamidino-2-phenylindole; LAD: ligation of left anterior coronary artery. Data analysis used two-way ANOVA followed by Bonferroni *post-hoc* tests for multiple comparisons (**C**,**E**,**F**,**G**,**H**,**I**). Data shown are mean ± SEM.

To further elucidate the possible mechanisms responsible for the repair of the aged myocardium, Sca-1^+^ and Sca-1^−^ chimeric mice received BrdU (5-bromo-2′-deoxyuridine) injections for 3 days to identify proliferating cells in the myocardium at the time point indicated (Fig. 4A). Cardiac proliferating cells were assessed 3 days post-MI (LAD3, Fig. 4A). The border and scar zones had the greatest numbers of BrdU^+^ cells and the number of proliferative cells in these regions was significantly greater in Sca-1^+^ than Sca-1^−^ chimeras (Fig. 4B and C). Of these proliferative cells, about 30% in the border zone and 44% in the scar region were homed donor progenitor cells in Sca-1^+^ chimeras (Fig. 4D and E), whereas almost all (92% and 95% in the border and scar zones, respectively) of the proliferative cells in Sca-1^−^ chimeras were host-cell derived (Fig. 4F), indicating that Sca-1^+^ cells had greater proliferative potential. Further analyses revealed that, of those proliferative cardiac-resident BM-derived progenitor cells, about 15% of cells were cKit-positive in Sca-1^+^ chimeras compared to only 3% in Sca-1^−^ chimeras (Fig. 4G). Flow cytometric analysis demonstrated that MI increased the number of cardiac-resident BM-derived progenitor cells in Sca-1^+^ chimeras (Fig. 4H), confirming the superior proliferative capability of Sca-1^+^ cells. Flow cytometry also showed that the number of GFP/Sca-1 double-positive cells in Sca-1^+^ chimeras decreased significantly 3 days post-MI (Fig. 4I) compared to baseline, although the number of GFP-positive cells increased dramatically after MI. This suggests the gradual loss of Sca-1 markers after myocardial injury, perhaps because of cell differentiation. Furthermore, proliferating cardiomyocytes were identified as both BrdU^+^ and actinin^+^ cells. As shown in Supplementary Fig. S4A, we did not find any newly formed cardiomyocytes. In addition, wheat germ agglutinin staining was used to evaluate changes in cardiomyocyte size. We did not find any changes in cardiomyocyte size between the Sca-1^+^ and the Sca-1^−^ groups (Supplementary Fig. S4B).

### Homed BM Sca-1^+^ cells rejuvenated aged heart through PDGFRβ pathway

**Figure 5:**
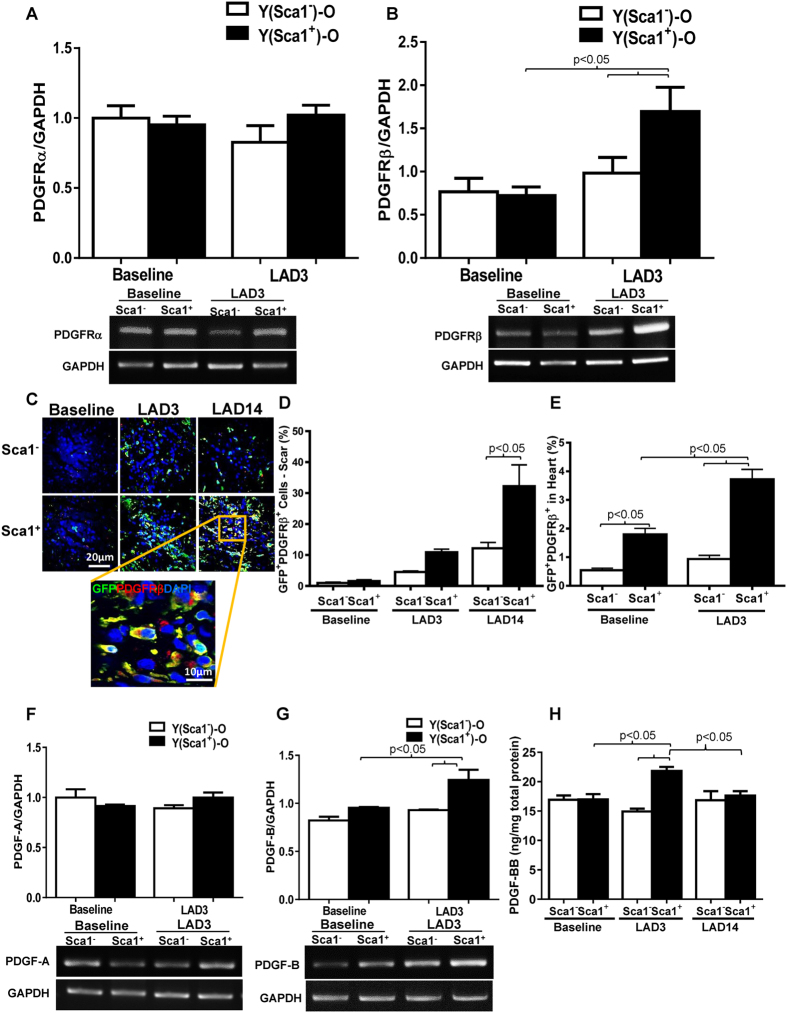
Homed BM Sca-1^+^ cells rejuvenated aged heart through PDGFRβ pathway. PDGFRα (**A**, n = 3–6/group) and PDGFRβ (**B**), n = 3/group) mRNA expression is depicted, quantified by RT–PCR 3 days post-myocardial infarction (MI) in Sca-1^+^ [(YSca1^+^)-O] and Sca-1^−^ [(YSca1^−^)-O] chimeric hearts. Myocardial staining 3 (LAD3) and 14 (LAD14) days post-MI for GFP/PDGFRβ double-positive cells **(C**). Quantification of GFP^+^/PDGFRβ^+^ cells by immunolabeling in the scar regions of infarcted hearts 3 and 14 days post-MI **(D**, n = 3/group). GFP^+^/PDGFRβ^+^ cells quantified in the whole heart by flow cytometry at baseline and 3 days post-MI **(E**, n = 3–4/group). PDGF-A **(F**, n = 3–6/group) and PDGF-B **(G**, n = 3/group) mRNA expression in the whole heart, quantified by RT–PCR 3 days post-MI. Total myocardial levels of PDGF-BB 3 and 14 days post-MI determined by Enzyme-linked Immunosorbent Assay [ELISA] **(H**, n = 3–5/group). LAD: ligation of left anterior coronary artery; PDGF: platelet-derived growth factor. Data analysis used two-way ANOVA followed by Bonferroni *post-hoc* tests for multiple comparisons (**A**,**B**,**D**,**E**,**F**,**G**,**H**). Data shown are mean ± SEM.

We evaluated Sca-1^+^ cell differentiation from 14 days up to 4 months after MI and found that myocardial injury did not initiate their vascular or cardiac differentiation (Supplementary Figs S5A,B and C) which is consistent with our previous findings^10^. RT-PCR analysis showed that there was no difference in the expression level of PDGFRα between the two groups 3 days post-MI (Fig. 5A); however, there was significantly increased expression of PDGFRβ in the infarct region of Sca-1^+^ compared to Sca-1^−^ chimeric hearts (Fig. 5B). Immunolabeling of myocardial sections for GFP and PDGFRβ or PDGFRα at 3 and 14 days (LAD14) post-MI identified multiple GFP/PDGFRβ double-positive cells (Fig. 5C) but very few, if any GFP/PDGFRα double-positive cells (data not shown) in infarcted hearts. Both immunofluoresent (Fig. 5D) and flow cytometric (Fig. 5E) quantification in the scar region and whole heart, respectively showed greater numbers of GFP/PDGFRβ double-positive cells in Sca-1^+^ than Sca-1^−^ chimeric hearts. The GFP/PDGFRβ double-positive cells persisted in the infarcted hearts for up to 4 months after MI (Supplementary Fig. S5D) though very few GFP^+^ cells expressed Sca-1 (Supplementary Fig. S5E). Consistent with the expression pattern of receptors, mRNA levels of PDGF-B, but not PDGF-A were significantly higher in the infarcted hearts of Sca-1^+^ compared with Sca-1^−^ chimeras (Fig. 5F and G). Quantification of the level of PDGF-BB homodimer by ELISA showed that only Sca-1^+^ chimeras responded to MI by increasing PDGF-BB homodimer (Fig. 5H).

### Homed BM Sca-1^+^ cells stimulated cell proliferation through activation of PDGFRβ-Akt/p27 Kip1 signaling

**Figure 6:**
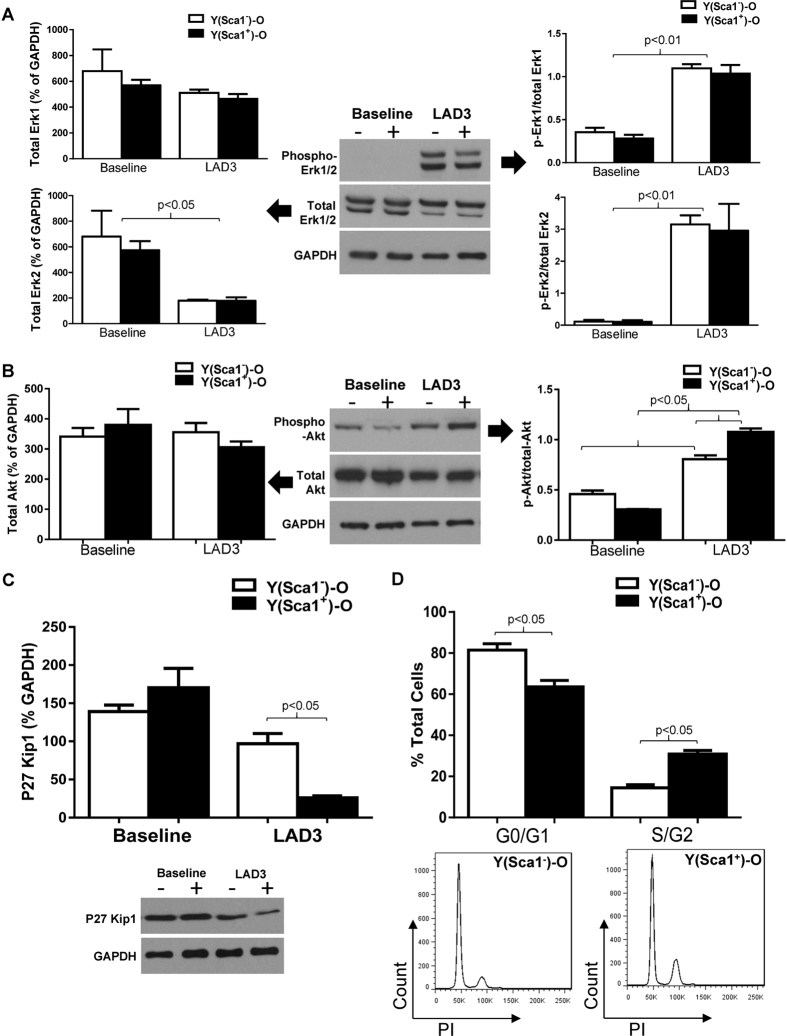
Homed BM Sca-1^+^ cells stimulated cell proliferation through activation of PDGFRβ -Akt/p27 Kip1 signaling. Phosphorylation of Erk1/2 and total Erk1/2 in the infarcted myocardium of Sca-1^+^ [(YSca1^+^)-O] and Sca-1^−^ [(YSca1^−^)-O] chimeras 3 days post-myocardial infarction (MI) is depicted with GAPDH used as a control **(A**, n = 3–7/group). Phosphorylation of Akt and total Akt in the infarcted myocardium 3 days post-MI [LAD3] (**B**, n = 3–7/group) with GAPDH used as a control. The level of p27Kip1 determined by Western blot and normalized by GAPDH in the infarcted myocardium 3 days post-MI (**C**, n = 3/group). Quantification of cells entering S/G2 phase by propidium iodide (PI) staining in the infarcted chimeric hearts 3 days post-MI **(D**, n = 3/group). GAPDH: glyceraldehyde 3-phosphate dehydrogenase; LAD: ligation of left anterior coronary artery. Data analysis used two-way ANOVA followed by Bonferroni *post-hoc* tests for multiple comparisons (**A**,**B**,**C**,**D**). Data shown are mean ± SEM.

RAS/MAPK signal pathway molecule expression was quantified in chimeric hearts in response to MI. The protein level of total ERK2 was decreased, but phospho-Erk1/2 was significantly increased in the hearts of both chimeras post-MI (Fig. 6A). No significant increase in the level of phospho-p44/42 Erk1/2 was found in the Sca-1^+^ compared with the Sca-1^−^ chimeras post-MI, suggesting that the RAS/MAPK signal pathway may not be involved in Sca-1-mediated cell proliferation. Conversely, the level of phospho-Akt was significantly elevated in Sca-1^+^ compared to Sca-1^−^ chimeric hearts post-MI, although the total Akt protein level was unchanged in both groups (Fig. 6B). In response to activation of Akt, expression of p27 Kip1, the downstream mediator of Akt, was significantly decreased in Sca-1^+^ compared to Sca-1^−^ chimeras (Fig. 6C). It has been suggested that activation of Akt and subsequent deactivation of p27 affects the G1 phase of the cell cycle. Indeed, propidium iodide staining for cell cycle detection showed that there were significantly more cells entering the S/G2 phase in the infarcted hearts of Sca-1^+^ compared to Sca-1^−^ chimeras (Fig. 6D). Collectively, these data suggest that the Sca-1 subset of BM cells activates the PDGFR-β-Akt/p27 pathway, thus affecting cell proliferation.

### Homed BM Sca-1^+^ cells stimulated angiogenesis after MI

**Figure 7:**
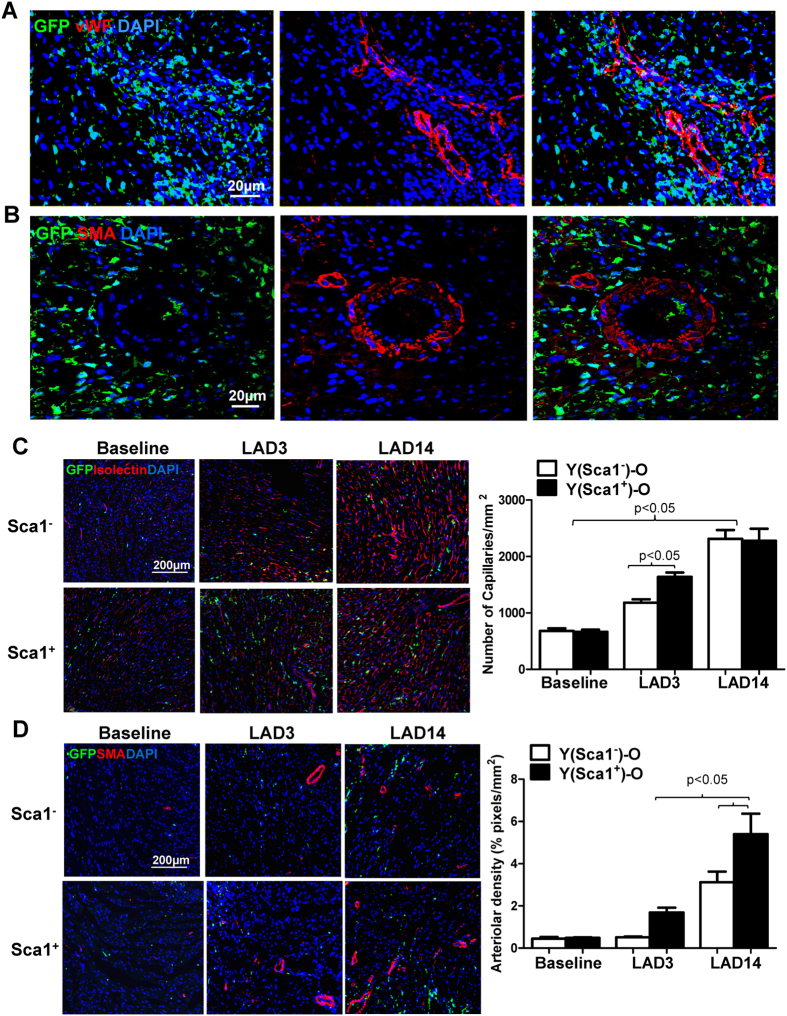
Homed BM Sca-1^+^ cells stimulated angiogenesis after MI. (**A**) Representative confocal images illustrating von Willebrand factor (vWF) immunolabeling of myocardial sections 14 days post-MI (LAD14) in Sca1^+^ chimeras. (**B**) Representative confocal images illustrating smooth muscle actin (SMA) immunolabeling of myocardial sections 14 days post-MI in Sca1^+^ chimeras. (**C**) Myocardial staining 3 (LAD3) and 14 days post-MI for isolectin B4 was employed to quantify the number of capillaries in Sca1^+^ and Sca1^−^ chimeric hearts (n = 3–4/group). (**D**) Myocardial staining 3 and 14 days post-MI for SMA was employed to quantify arteriolar density in Sca1^+^ and Sca1^−^ chimeric hearts (n = 3/group). LAD: ligation of left anterior coronary artery. Data analysis used two-way ANOVA followed by Bonferroni *post-hoc* tests for multiple comparisons (**C**,**D**). Data shown are mean ± SEM.

To understand how the homed BM Sca-1^+^ cells mediated cardiac repair, we evaluated cellular angiogenesis after MI. Immunolabeling of endothelial and smooth muscle cells was conducted by von Willebrand factor (vWF) and smooth muscle actin (SMA) staining, respectively. Fourteen days after MI, the BM Sca-1^+^ cells were located in high densities near capillary structures (Fig. 7A) or arteriolar structures (Fig. 7B) though they did not differentiate into endothelial or smooth muscle cells. Histological analysis demonstrated that capillary density according to isolectin staining was significantly greater in Sca-1^+^ than Sca-1^−^ chimeric hearts especially at an early stage of MI (LAD3, Fig. 7C). Arteriolar density was also significantly greater in Sca-1^+^ than Sca-1^−^ chimeric hearts (Fig. 7D). Interestingly, the increased arteriolar density was sustained up to 14 days after MI, suggesting improved arteriogenesis in the Sca-1^+^ chimeric hearts. These results suggest that Sca-1^+^ cells may induce paracrinal support and repair through improved angiogenesis during myocardial recovery.

## Discussion

We found that Sca-1^+^ cells are a key BM cell type involved in the rejuvenation of the aged heart. Following whole BM reconstitution of lethally-irradiated old mice, more young Sca-1^+^ cells homed to the aged heart than other BM cell types. When we reconstituted old mice with Sca-1^+^ or Sca-1^−^ cells, Sca-1^+^ chimeras had better restoration of myocardial progenitors which were stably integrated within the myocardium twelve weeks after reconstitution. Since young Sca-1^+^ cells homed to the aged myocardium without heart injury, these cells may be capable of rejuvenating the aged heart.

Granulation tissue formation and cell proliferation are critical steps in myocardial repair after an MI. Virag *et al*. found that myofibroblast and endothelial cell proliferation shows an abrupt peak 4 days post-MI, decreases by 1 week, and effectively ceases by 14 days post-MI^26^. This active cell proliferation in the early phase of an MI results in increased vessel area and effective scar contraction of the infarcted heart in the later phase of MI. However, in the myocardium of an aged individual, cellular proliferation is diminished likely due to the presence of fewer intrinsic cardiac stem cells that are characterized by compromised proliferative capacity. Reduced proliferation negatively impacts tissue repair which can lead to regional dilation and thinning of the infarct zone after an injury.

In the current study, our major finding was that in response to injury, Sca-1^+^ cells were able to home to the aged heart to repopulate the stem cell population in the tissue. These cells not only actively proliferated to repair injured tissue after an MI, but also stimulated the proliferation of host cells for tissue repair to a greater extent than Sca-1^−^ cells. Of these proliferative cells, there were significantly more donor-derived progenitor cells in the Sca-1^+^ chimeras than in the Sca-1^−^ chimeras indicating that Sca-1^+^ cells had superior proliferative potential. On the other hand, the proliferation of host cells in the border and infarct zones were also significantly greater in Sca-1^+^ than Sca-1^−^ chimeras. Indeed, we found that there were significantly more cells entering the S/G2 phase in the infarcted hearts of Sca-1^+^ than Sca-1^−^ chimeras, indicating an overall increase in the number of cells entering a proliferative state in Sca-1^+^ chimeric hearts. Collectively, these data suggest that the Sca-1^+^ subset of BM cells activates the cell proliferation pathway and the regenerative activation of both homed donor and host cells following reconstitution with Sca-1^+^ cells improved healing of the injured aged heart, just as occurred following whole BM cell reconstitution^10^. Sca-1^+^ chimeras had smaller infarct areas and thicker infarct tissue due to an increase in cellular proliferation. This improved healing process resulted in Sca-1^+^ chimeric mice having better load-dependent indices of ventricular cardiac function as well as less extensive infarct scars and increased blood vessel density, suggesting that Sca-1^+^ cells provided paracrinal support and repair during myocardial recovery. Other studies have also demonstrated that the number of Sca-1^+^ cells increased in the heart^24^ and contributed to regeneration and repair after MI^25^. Those studies also showed that injection of Sca-1^+^ cardiac stem cells into infarcted hearts attenuated infarct size and preserved left ventricular function^24^,^27^.

Further analysis revealed that there were more BM-derived cells which co-expressed PDGFRβ in Sca-1^+^ than Sca-1^−^ chimeric hearts, and the number of GFP/PDGFRβ double-positive cells increased in Sca-1^+^ but not Sca-1^−^ chimeric hearts after MI. Accordingly, only Sca-1^+^ chimeras responded to MI by increasing PDGF-BB homodimers. These results suggest that the effects of Sca-1^+^ cells may be mediated at least in part through the PDGFRβ pathway. It has been suggested that PDGFR-β activates the RAS/MAPK signal pathway molecules, K-RAS, p-Raf1, p-MEK1, and p-ERK1/2, thus inducing cell proliferation^28^,^29^,^30^,^31^. It has been shown that in granulopoietic precursors, Sca-1 expression positively correlated with ERK activation, and a lack of Sca-1 led to ERK inactivation^32^. However, our data revealed that Sca-1 negatively regulated ERK activation in infarcted hearts. This finding is consistent with a recent study which showed that Sca-1 overexpression negatively regulated ERK activation in a hypertrophic heart^21^.

Akt is a critical signal pathway in heart development. However, long-term activation of Akt causes pathological hypertrophy and heart failure^33^. Here we showed that Sca-1 activated Akt possibly through the PDGFR-β pathway. The subsequent de-activation of P27 by phospho-Akt led to an increase in cell cycle activity in the S/G2 phase and eventually stimulated overall cell proliferation. Cell proliferation in both the homed BM cells and the host progenitors may be a major contributor to the functional recovery of the aged heart. In contrast to our finding, it has been shown that Sca-1 disruption enhanced Akt activation induced by aortic banding, whereas Sca-1 overexpression inhibited phosphorylation of cardiac Akt, indicating that Sca-1 is a negative regulator of Akt^21^. A potential reason for the discrepancy in results is that the effect of Sca-1 on Akt activation may depend on the cell, tissue, and disease background. Our data revealed that Sca-1 could protect against MI via regulation of the PDGFR-β-Akt signaling pathways. However, the involvement of PDGFR-β/Akt/p27 signaling in the Sca-1 treatment effect was observational and more conclusive evidence is required. A future study using PDGFR-β knock-out (KO) mice^34^ should be performed using BM reconstitution with young BM Sca-1 cells followed by MI. Functional analysis as well as a mechanistic dissection of the PDGFR-β/Akt/p27/cell proliferation pathway should be evaluated. We postulate that the functional benefit of BM Sca-1 cells will be lost and activation of the PDGFR-β/Akt/p27 pathway will be blocked in PDGFR-β KO mice reconstituted with young BM Sca-1 cells.

We identified a specific BM cell type, Sca-1^+^, and demonstrated that these cells are important contributors to the repair of ventricular function in the aged heart. Sca-1^+^ cells may be an important new target in aged tissue rejuvenation/regeneration to prevent progressive heart failure in the growing number of aged patients.

## Methods

Detailed methodology is provided in the Supplementary Material.

## Study 1

### Experimental Animals and Whole Bone Marrow Reconstitution

The Animal Care Committee of the University Health Network approved all experimental procedures, which were carried out according to the *Guide for the Care and Use of Laboratory Animals* (NIH, 8^th^ Edition, 2011). Both male and female animals were used and mice were randomly assigned to groups. A power analysis determined the number of animals to include in each group. The investigator was blinded to the experimental condition. Mice were sacrificed with an overdose of 5% isoflurane (Pharmaceutical Partners of Canada Inc., Richmond Hill, ON, Canada) via the endotracheal route which was followed by exsanguination.

Whole BM reconstitution was used to identify the cell type most beneficial for cardiac rejuvenation. C57BL/6 mice aged 20–22 months (old recipients, n = 22) were lethally irradiated (10.5 Gy) and immediately received an infusion (through the tail vein) of fresh BM cells (5 × 10^6^) from either young (2–3 months, n = 4) or old (20–22 months, n = 4) green fluorescence protein (GFP)-positive mice (C57BL/6-Tg-GFP mice, The Jackson Laboratory). Infusion of BM cells from young mice generated young chimeras (Y-O), while infusion of BM cells from old mice generated old chimeras (O-O). Mice were sacrificed 12 weeks after BM reconstitution for immunofluorescent staining and flow cytometric analysis (see methodology below) to identify homed BM progenitors in the aged heart at steady state.

## Study 2

### Experimental Animals and Sca-1^+^ or Sca-1^−^ Bone Marrow Reconstitution

For BM reconstitution with Sca-1^+^ or Sca-1^−^ cells, BM was flushed from the tibias and femurs of C57BL/6-Tg-GFP mice aged 2–3 months (n = 80), and mononuclear cells were separated by density gradient centrifugation, then separated into positively- and negatively-labeled fractions by immunomagnetic activated cell sorting following the manufacturer’s instructions (Cat#: 18756, STEMCELL Technologies, Vancouver, BC, Canada). The purity of positive cells was confirmed by flow cytometry. Sca-1 antigen-positive or antigen-negative cells (2 × 10^6^) were mixed with 1 × 10^5^ old BM supporting cells and injected into lethally-irradiated C57BL/6 mice aged 20–22 months (old recipients, n = 240 in total) via the tail vein to create Sca-1^+^ [Y(Sca1^+^ )-O] and Sca-1^−^ [Y(Sca1^−^)-O] chimeras, respectively. A control group (Old) of aged C57BL/6 mice (aged 20–22 months) was used for the functional analyses.

### Myocardial infarction and cardiac function measurement

Twelve weeks after Sca-1^+^ or Sca-1^−^ cell BM reconstitution, coronary artery occlusion was performed as previously reported^35^. Briefly, mice were intubated and ventilated with 2% isoflurane. Through a left thoracotomy, the pericardium was entered and the left anterior descending coronary artery was ligated. MI was confirmed by echocardiography 3 and 7 days after ligation. To minimize variation, only those mice with fractional shortening between 20 and 40% were kept in the study. Cardiac function was measured with echocardiography at different time points before and after MI and with a pressure–volume catheter at the end of the study. At study end, scar tissue was identified with Masson’s trichrome, and scar area and thickness were measured by planimetry. Scar size was presented as a percentage of the left ventricular wall (LVW) length at every heart section and calculated as follows: (epicardial scar length)/(epicardial LVW length) + (endocardial scar length)/endocardial LVW length).

### Immunofluorescent staining and confocal microscopy

Hearts were fixed in 2% paraformaldehyde for 24 h after being adequately flushed with PFA. They were then placed in 0.5 M sucrose at 4 °C overnight. Hearts were then embedded with optical cutting temperature (OCT) compound, and 5-μm-thick frozen sections were prepared. For immunofluorescent staining, the slides were incubated with the primary antibodies at room temperature for 2 h. Incubation with respective Alexa 488 or 568 or 647 conjugated secondary antibodies was carried out at room temperature with light protection for 1 h. The nuclei were identified with 4′,6-diamidino-2-phenylindole (DAPI). The number of positive cells in five randomly selected high-power fields per section was determined with a Nikon Eclipase Ti fluorescent microscope. An Olympus Fluoview 2000 laser scanning confocal microscope was used to confirm the co-localization of fluorescent signals. To determine cell proliferation after MI, 5-bromo-2′-deoxyuridine (BrdU) was administered to the mice by intraperitoneal injection (50 mg/kg) for 3 consecutive days. Coronary artery ligation was performed 1 day later. Animals were sacrificed 3 days after ligation and hearts were excised. Frozen tissue sections (10 μm) were stained for BrdU incorporation.

### Protein Isolation, Enzyme-linked Immunosorbent Assay (ELISA) and Western Blotting

To determine myocardial protein levels, the heart samples were separated in the scar and remote (normal muscle) regions and homogenized in liquid nitrogen. The total protein was extracted from powdered tissue in lysis buffer. The level of platelet-derived growth factor-BB (PDGF-BB) was determined using ELISA following the manufacturer’s instructions and normalized by ng/mg total protein.

For Western Blotting, 50 μg of lysate was fractionated through a 4% stacking and 10% running SDS-PAGE gel, and the fractionated proteins were transferred to a polyvinylidene difluoride (PVDF) membrane. Blots were blocked for 1 h at room temperature with blocking buffer. The primary antibodies were reacted with the blots overnight at 4 °C. After three washings, the blots were incubated with horseradish peroxidase-conjugated secondary antibody for 1 h at room temperature. Visualization was performed with enhanced chemiluminescence. For quantification, densitometry of the target bands was divided by the corresponding densitometry of the glyceraldehyde 3-phosphate dehydrogenase (GAPDH) band using AlphaImager 2200 software (ProteinSimple, San Jose, CA, USA).

### RNA extraction, RT-PCR

Total RNA was isolated from mouse hearts using Trizol reagent (Sigma). The heart samples were separated into the scar and remote (normal muscle) regions and homogenized in liquid nitrogen. One hundred mg of ground tissue was taken for total RNA extraction. Reverse transcription was performed using SuperScript III (Invitrogen) and 1 μg of total RNA served as the template for each reaction. The RT-PCR products were separated on 1% agarose gel containing ethidium bromide. Mouse GAPDH served as a loading control. The sequences of the mouse primers used in RT-PCR are shown in Supplementary Table S1.

### Flow Cytometry Analysis

Mouse hearts were collected 3 days post-MI, and separated into the injured and non-injured segments prior to digestion using 0.1% collagenase type II at 37 °C for 30 min. All antibody incubations were carried out for 30 min at 4 °C in the dark. Cells were analyzed using an FC500 flow cytometer (Beckman Coulter). The fluorescence intensity of 10,000 cells for each sample was quantified.

For analysis of cell cycle, the cells from the digested mouse hearts were washed twice with PBS, suspended in 75% ethanol, and fixed by incubation in 75% ethanol at 4 °C for 2 h. Fixed cells were collected by centrifugation, washed with PBS, treated with RNase (500 ng/ml), and stained with propidium iodide (40 μg/ml) at 4 °C for 3 h. A fluorescence-activated cell sorting (FACS) flow cytometer (BD Biosciences, USA) was used to determine cellular DNA contents. The percentage of cells in G0/G1, S, and G2/M phases was determined using the Cell FIT Cell Cycle Analysis software (version 2.01.2; BD Biosciences).

### Statistical analysis

All values are expressed as mean ± SEM. Analyses were performed using GraphPad Prism 6.0 software. Data were normally distributed and variance was similar between groups being statistically compared. Student’s *t*-test was used for two-group comparisons. Comparisons of parameters among three groups were analyzed using one-way analysis of variance (ANOVA) followed by Tukey *post-hoc* tests when the F value of the ANOVA was significant or two-way ANOVA for two-factor variables followed by Bonferroni *post-hoc* tests. Fractional shortening was analyzed by one-way repeated-measures ANOVA followed by Bonferroni *post-hoc* tests. Differences were considered statistically significant at *p* < 0.05.

## Additional Information

**How to cite this article**: Li, S.-H. *et al*. Young Bone-Marrow Sca-1^+^ Stem Cells Rejuvenate the Aged Heart and Improve Function after Injury through PDGFRβ-Akt pathway. *Sci. Rep.* **7**, 41756; doi: 10.1038/srep41756 (2017).

**Publisher's note:** Springer Nature remains neutral with regard to jurisdictional claims in published maps and institutional affiliations.

## Supplementary Material

Supplementary Material
