# Supplementary material for: Young Bone-Marrow Sca-1+ Stem Cells Rejuvenate the Aged Heart and Improve Function after Injury through PDGFRβ-Akt pathway
Source: Sci Rep. 2017 Jan 31;7:41756. doi: 10.1038/srep41756 (PMC5282531; doi:10.1038/srep41756)
Supplement: Supplementary Material [file srep41756-s1.pdf]

Supplementary Material

for

Young Bone-Marrow Sca-1$^+$ Stem Cells Rejuvenate the Aged Heart and Improve Function after Injury through PDGFRβ-Akt pathway

Shu-Hong Li, MD, MSc[1,#]; Lu Sun, MD, PhD[1,3,#]; Lei Yang, MD, PhD[1,3]; Jiao Li, MD, PhD[1,4]; Zhengbo Shao, MD, PhD[1,3]; Guo-Qing Du, MD, PhD[1,3]; Jun Wu, MD, MSc[1]; Richard D. Weisel, MD[1,2]; Ren-Ke Li, MD, PhD[1,2,*]

[1]Division of Cardiovascular Surgery, Toronto General Research Institute, University Health Network, Toronto, ON, Canada

[2]Division of Cardiac Surgery, Department of Surgery, University of Toronto, Toronto, ON, Canada

[3]Research Institute, Second Affiliated Hospital of Harbin Medical University, Harbin, China

[4]Department of Cardiology, Second Affiliated Hospital of Guangzhou Medical University, Guangzhou Institute of Cardiovascular Disease, Guangzhou, China

[#]These authors contributed equally to this work.

Supplementary Methods

Immunofluorescent staining and confocal microscopy

Hearts were fixed in 2% paraformaldehyde [(PFA), Sigma, Oakville, ON, Canada] for 24 h after being adequately flushed with PFA. They were then placed in 0.5M sucrose at 4°C overnight. Hearts were then embedded with optical cutting temperature (OCT) compound, and 5-μm-thick frozen sections prepared. The sections were dried at room temperature briefly, followed by fixation in 2% PFA for 10 min at 4°C. For immunofluorescent staining, the slides were incubated with one of the following primary antibodies: Alexa 488 conjugated anti-GFP (Cat#: A21311, Invitrogen) or Goat anti-GFP (Cat#: AB6673, Abcam), anti-Sca-1 (Cat#: AF1226, R&D system), anti-CD14 (Cat#: 553738), 34 (Cat#: 553731), 45 (Cat#: 550539), PDGFRα (Cat#: 558774) and cKit (Cat#: 553352, all from BD Biosciences), anti-Flt-1 (Cat#: V4262), α smooth muscle actin (α-SMA, Cat#: A2547) and sarcomeric actinin (SARC, Cat#: A7811, all from Sigma), anti-CD11b (Cat#: 14-0112, eBiosciences), anti-PDGFR β (Cat#: SC-432, Santa Cruz), anti-von Willebrand factor (vWF, Cat#: A0082, Dako) at room temperature for 2 h. Incubation with respective Alexa 488 or 568 or 647 conjugated secondary antibodies (Invitrogen) was carried out at room temperature with light protection for 1 h. The nuclei were identified with 4',6-diamidino-2-phenylindole [(DAPI), Cat#: D-6883, Sigma, Oakville, ON, Canada]. For evaluation of the number of capillaries, Alexa 568 conjugated isolectin B4 (Cat#: I21412, Invitrogen) staining was carried out at a 1:100 dilution for 1h at room temperature. The number of positive cells in five randomly selected high-power fields per slide was determined and averaged with a Nikon Eclipase Ti fluorescent microscope. The number of animals used for

each quantification is indicated in the figure legends. An Olympus Fluoview 2000 laser scanning confocal microscope was used to confirm the co-localization of fluorescent signals.

To determine cardiomyocyte size, Alexa 555 conjugated wheat germ agglutinin (WGA, Invitrogen, Cat#: W32464) staining was carried out at a 1:200 dilution for 2 h at RT for the cross heart sectional areas. The area size of cardiomyocytes was measured in 6 distinct microscope fields from each slide using ImageJ software (National Institutes of Health, Bethesda, MD, USA). Five hearts were analyzed for each experimental group.

To determine cell proliferation after MI, 5-bromo-2'-deoxyuridine [(BrdU), 50 mg/kg; Cat#: B5002, Sigma] was administered to the mice by intraperitoneal injection for 3 consecutive days. Coronary artery ligation was performed 1 day later. Animals were sacrificed 3 days after ligation and hearts were excised. Frozen tissue sections (10 µm) were stained for BrdU incorporation. Briefly, cryosections were fixed in 2% PFA. Tissue sections were first treated with 2N HCl for 30 min (10 min at RT, 20 min at 37°C) to expose the antigens and then permeabilized with 0.5% Triton X-100. Sections were incubated with rat anti-BrdU (1:80; Cat#: AB6326, Abcam) for 2 h in a humidified atmosphere. After incubation with the primary antibody, the slides were washed three times in phosphate buffered saline (PBS) and incubated with an Alexa 568-conjugated goat anti-rat secondary antibody (Cat#: A-11077, Invitrogen) for 1 h at room temperature. Tissue sections were subsequently stained with the anti-GFP antibody. The nuclei were identified with DAPI staining. Labelled nuclei cells were quantified in 10 independent fields by microscopic analysis (NiKon), with the BrdU-labeling index expressed as the ratio of BrdU^{+} cells or BrdU^{+}/GFP^{+} cells to total nuclei.

We used ImageJ software to quantify the number of capillaries in the isolectin B4-positive area. In the SMA-stained sections, SMA-positive blood vessels (≥30 μm) were identified and quantified.

Protein Isolation, Enzyme-linked Immunosorbent Assay (ELISA) and Western Blotting

To determine myocardial protein levels, the heart samples were separated in the scar and remote (normal muscle) regions and homogenized in liquid nitrogen. The total protein was extracted from powdered tissue in lysis buffer (20 mM Tris [pH7.4], 150 mM NaCl, 1mM EDTA, 1mM EGTA, 1% Triton, 2.5 mM Na pyrophsphate, 1mM β-glycerolphosphate, 1 mM Na_3VO_4, 1 μg/ml of leupeptin, 1μg/ml of pepstatin, and 1 mM phenylmethylsulfonyl fluoride) for 1 h on ice. After centrifugation at 10,000 *g* for 10 min, the supernatant was collected and protein concentration was determined using a Bio-Rad DC protein assay kit. The level of platelet-derived growth factor-BB [(PDGF-BB), Cat#: MBB00, R&D system] was determined using ELISA following the manufacturer's instructions and normalized by ng/mg total protein.

For Western Blotting, 50 μg of lysate was fractionated through a 4% stacking and 10% running SDS-PAGE gel, and the fractionated proteins were transferred to a polyvinylidene difluoride (PVDF) membrane. Blots were blocked for 1 h at room temperature with blocking buffer. The antibodies (rabbit anti-p44, 42-Erk1/2, Cat#: 9101, total Erk1/2, Cat#: 9107, p-ser473-Akt, Cat#: 9271S, total Akt, Cat#: 4691, P27Kip1, Cat#: 3688S, all from Cell Signaling Technology, Danvers, MA, USA) were reacted with the blots overnight at 4°C. After washing (3× 5 min in 1× tris-buffered saline [TBS]-0.1% Tween 20), the blots were incubated with horseradish peroxidase-conjugated secondary antibody at 1:2000 dilution for 1 h at room temperature. Visualization was performed with enhanced chemiluminescence. For quantification,

densitometry of the target bands was divided by the corresponding densitometry of the GAPDH (Cat#: MAB374, Millipore) band using AlphaImager 2200 software (ProteinSimple, San Jose, CA, USA).

RNA extraction, RT-PCR

Total RNA was isolated from mouse hearts using Trizol reagent (Sigma). The heart samples were separated into the scar and remote (normal muscle) regions and homogenized in liquid nitrogen. One hundred mg of ground tissue was taken for total RNA extraction. Reverse transcription was performed using SuperScript III (Invitrogen) and 1 μg of total RNA served as the template for each reaction. Reverse transcription was performed at 50°C for 60 min. For PCR amplification of mRNA, samples were heated at 95°C for 5 min, followed by 30 cycles of 95°C for 30 s, 55°C for 1 min, 72°C for 1 min, and 72°C for an extra 10 min at the end of amplification. The RT-PCR products were separated on 1% agarose gel containing ethidium bromide. Mouse Glyceraldehyde 3-phosphate dehydrogenase (GAPDH) served as a loading control. The sequences of the mouse primers used in RT-PCR are shown in Supplementary Table 1.

Flow Cytometry Analysis

Mouse hearts were collected 3 days post-MI, and separated into the injured and non-injured segments prior to digestion using 0.1% collagenase type II (Worthington Biochemical Corportion, Lakewood, NJ, USA) at 37°C for 30 min. After filtering through a 70 μm filter, the cells were resuspended in PBS supplemented with 2% fetal bovine serum (FBS) and 0.1%

sodium azide. One million cells were taken for antibody staining with Alexa488 conjugated anti-GFP (Cat#: A21311, Invitrogen) or Goat anti-GFP (Cat#: AB6673, Abcam), anti-Sca-1 (Cat#: AF1226, R&D system), anti-PDGFR β (Cat#: SC-432, Santa Cruz). All antibody incubations were carried out for 30 min at 4°C in the dark. Alexa fluor 546-conjugated donkey anti-rabbit (Cat#: A-10040) or anti-goat (Cat#: A-11056, all from Invitrogen) was added for staining with mouse PDGFR β or Sca-1, respectively. Isotype-identical reagents served as controls (Becton Dickinson). Cells were analyzed using an FC500 flow cytometer (Beckman Coulter). The fluorescence intensity of 10,000 cells for each sample was quantified.

For analysis of cell cycle, the cells from the digested mouse hearts were washed twice with PBS, suspended in 75% ethanol, and fixed by incubation in 75% ethanol at 4 °C for 2 h. Fixed cells were collected by centrifugation, washed with PBS, treated with RNase (500 ng/ml; Sigma, USA), and stained with propidium iodide (40 µg/ml; Cat#: P4864, Sigma, USA) at 4 °C for 3 h. A fluorescence-activated cell sorting (FACS) flow cytometer (BD Biosciences, USA) was used to determine cellular DNA contents. The percentage of cells in G0/G1, S, and G2/M phases was determined using the Cell FIT Cell Cycle Analysis software (version 2.01.2; BD Biosciences).

Supplementary Table S1. Sequences of mouse primers used in RT-PCR.

Reverse Transcription PCR primers

Gene	Forward	Reverse
PDGFRα	GGGGACAGACTGTGAGGTGT	CAATCACCAACAGCACCAAC
PDGFRβ	CACCTTCTCCAGTGTGCTGA	CCATGGTCGTTCACACTCAC
PDGFA	GAGATACCCCGGGAGTTGAT	GATGGTCTGGGTTCAGGTTG
PDGFB	TATGAAATGCTGAGCGACCA	GGCTTCTTTCGCACAATCTC
GAPDH	CGGCACAGTCAAGGCCGAGAATGG	TCATGGATGACCTTGGCCAGGGGG

Primers used for Reverse Transcription (RT)-PCR of PDGFRα, PDGFRβ, PDGF-A, PDGF-B and GAPDH quantification.

Supplementary Figures

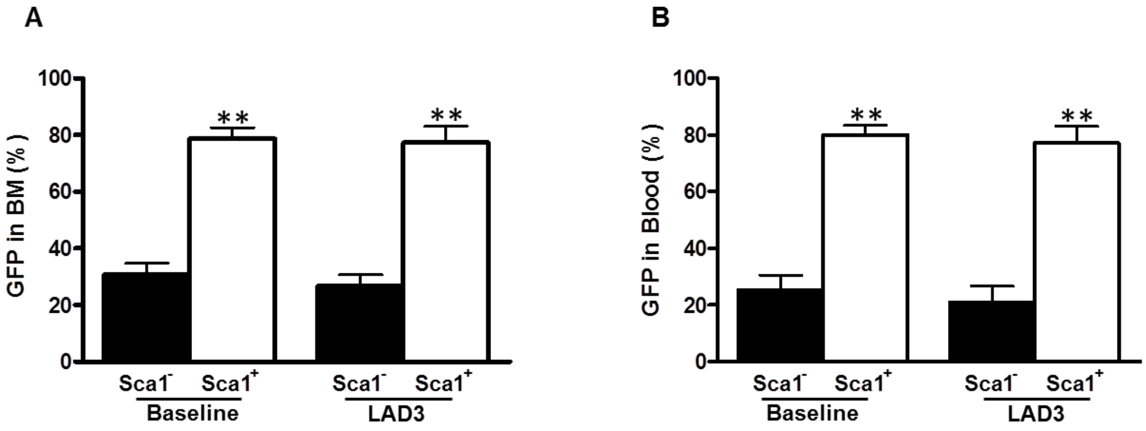

Supplementary Figure S1. Number of progenitor cells in chimeric mice after BM reconstitution.

The bone marrow (BM) of irradiated old mice was reconstituted with Sca-1$^+$ or Sca-1$^-$ cells from young donors, generating Sca-1$^+$ and Sca-1$^-$ chimeras. Twelve weeks later, GFP$^+$ cells in the BM (A, n = 3-7/group) and blood (B, n = 3-5/group) of the reconstituted mice were quantified by flow cytometry before and after MI. The number of GFP$^+$ cells in all the organs was significantly higher in Sca-1$^+$ compared to Sca-1$^-$ chimeras. $^{**}p<0.01$; GFP = green fluorescence protein. Data analysis used two-way ANOVA followed by Bonferroni *post-hoc* tests for multiple comparisons. Data shown are mean±SEM.

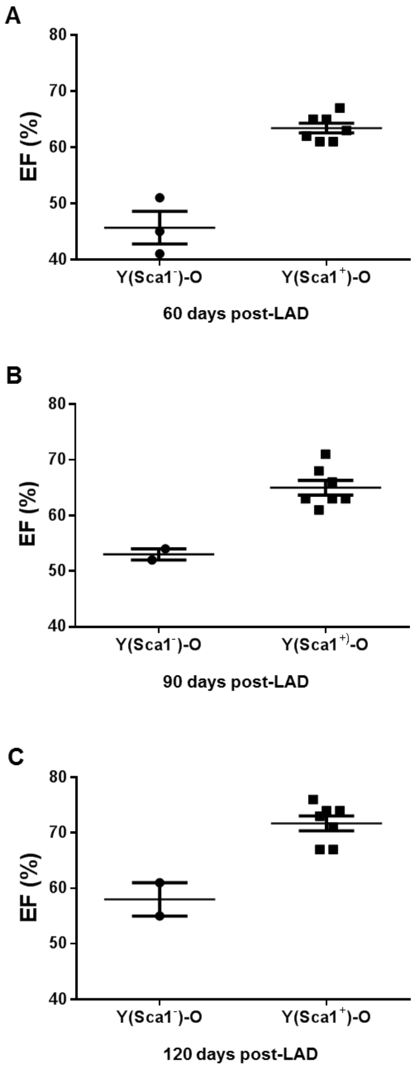

Supplementary Figure S2. Improved ventricular function in Sca-1$^+$ chimeras 3 months post-MI.

Myocardial infarction (MI) was induced in Sca-1$^+$ [(YSca1$^+$)-O] and Sca-1$^-$ [(YSca1$^-$)-O] chimeric mice 12 weeks after bone marrow (BM) reconstitution with Sca-1$^+$ or Sca-1$^-$ cells. Ejection fraction (%) was evaluated by serial echocardiography (n = 2-7/group) 60 **(A)** 90 **(B)**, and 120 **(C)** days post-MI. Data analysis used un-paired *t*-tests (A). Data shown are mean±SEM.

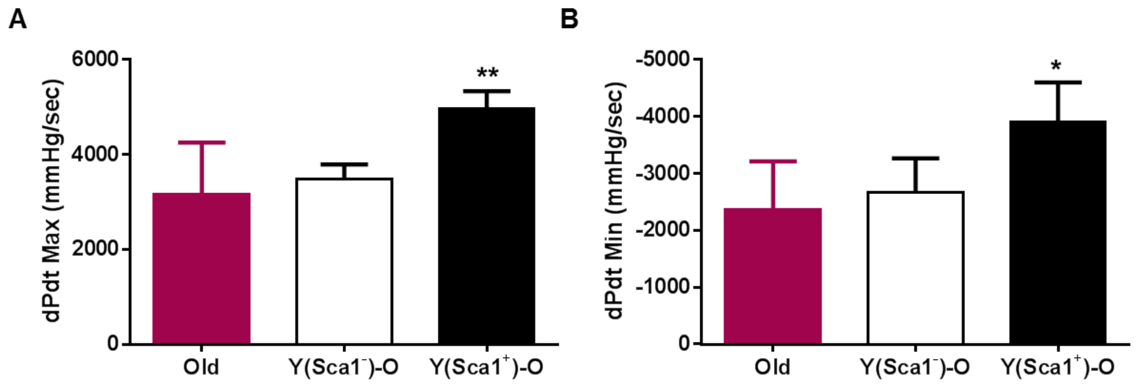

Supplementary Figure S3. Improved load-dependent indices of ventricular function in Sca-1^+ chimeras.

Myocardial infarction (MI) was induced in Sca-1^+ [(YSca1^+)-O] and Sca-1^- [(YSca1^-)-O] chimeric mice as well as in aged control mice (Old). Invasive haemodynamic measurements taken 4 weeks post-MI were used to calculate load-dependent indices of ventricular function, including dP/dt maximum **(A)** and dP/dt minimum **(B)**. n = 4-6/group; *p<0.05, **p<0.01. Data analysis used one-way ANOVA followed by Tukey *post-hoc* tests to evaluate differences among groups. Data shown are mean±SEM.

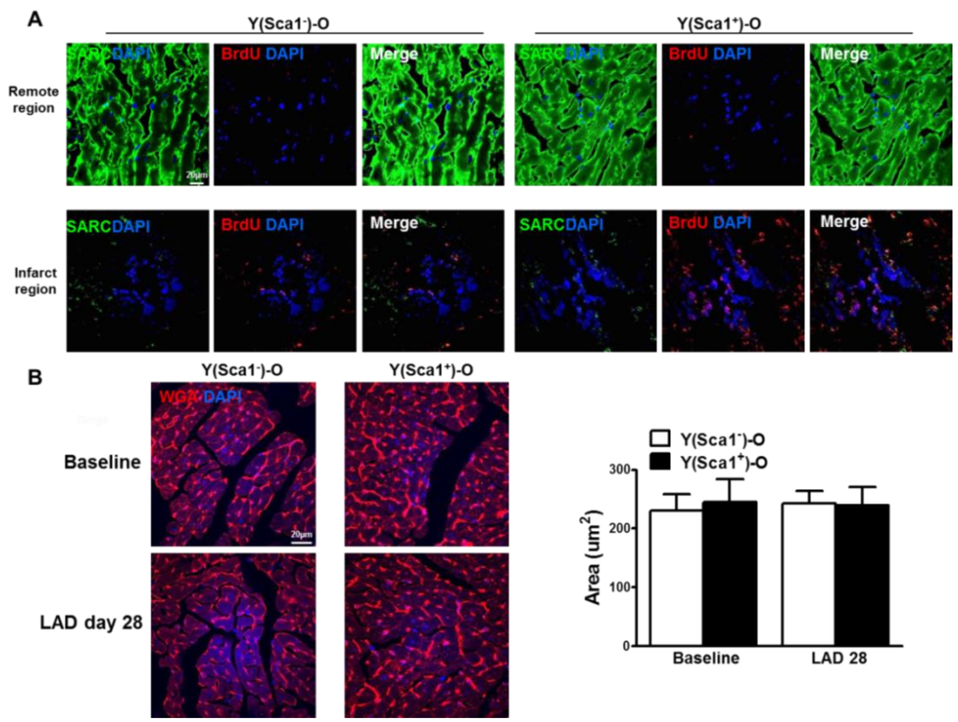

Supplementary Figure S4. **Formation of new cardiomyocytes and changes in cardiomyocyte size.**

Coronary artery ligation was performed 12 weeks after the bone marrow (BM) reconstitution of old recipients with young Sca-1^+ or Sca-1^- cells, and BrdU was given 1 day prior to and 2 days after myocardial infarction (MI). Cardiac proliferating cells were assessed 14 days post-MI. **(A)** Immunolabeling of myocardial sections permitting identification of cells which were either cardiac sarcomeric actinin [SARC]$^+$ (cardiomyocytes; green) and/or BrdU$^+$ (proliferating cells; red). Nuclei are stained blue with DAPI. No newly formed cardiomyocytes were found. **(B)** Wheat germ agglutinin (WGA) staining was used to evaluate changes in cardiomyocyte size. There were no changes in cardiomyocyte size between the [(YSca1^+)-O] and the [(YSca1^-)-O] groups at 28 days post-MI. BrdU: 5-bromo-2'-deoxyuridine; DAPI: 4’, 6-diamidino-2-phenylindole.

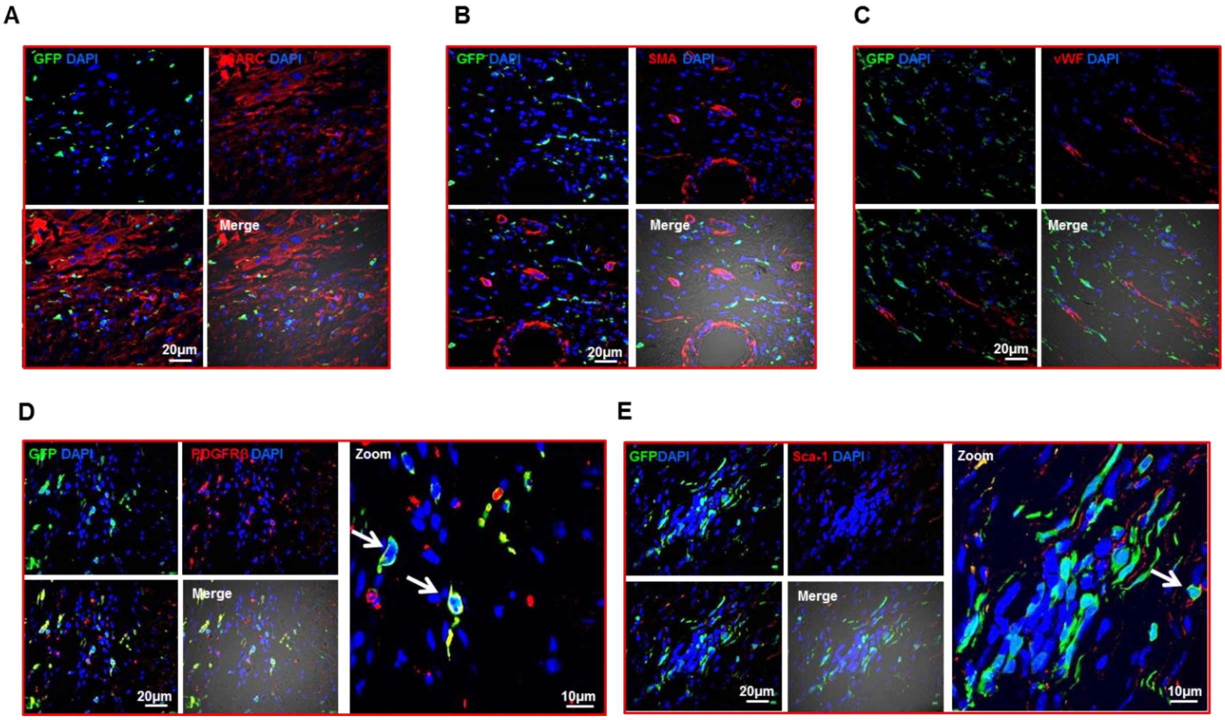

Supplementary Figure S5. Sustained homing of BM Sca-1 cells as perivascular PDGFRβ$^+$ cells in the infarcted myocardium.

Bone marrow of irradiated old mice was reconstituted with 2 × 10^6 bone marrow Sca-1$^+$ or Sca-1$^-$ cells from young donors (GFP, green), generating Sca-1$^+$ [(YSca1$^+$)-O] and Sca-1$^-$ [(YSca1$^-$)-O] chimeras, respectively. The coronary artery was ligated 12 weeks later. Immunolabeling of myocardial sections 4 months post-MI assessed progenitor cell differentiation in Sca-1$^+$ and Sca-1$^-$ chimeras. Representative confocal images show that GFP$^+$ cells were mostly negative for cardiac sarcomeric actinin **(**SARC, **A)**, smooth muscle actin **(**SMA, **B)**, and von Willebrand factor (vWF, **C**). Many GFP$^+$ cells were PDGFRβ$^+$ **(D)**, but few were Sca-1$^+$ **(E)**. GFP = green fluorescence protein; DAPI: 4’,6-diamidino-2-phenylindole.
